# Unravelling the inhibitory activity of *Chlamydomonas reinhardtii* sulfated polysaccharides against α-Synuclein fibrillation

**DOI:** 10.1038/s41598-018-24079-7

**Published:** 2018-04-09

**Authors:** Sinjan Choudhary, Shreyada N. Save, Sirisha L. Vavilala

**Affiliations:** UM-DAE Centre for Excellence in Basic Sciences, University of Mumbai, Kalina Campus, Mumbai, 400098 India

## Abstract

α-Synuclein (α-Syn) is an intrinsically disordered presynaptic protein, whose aggregation is critically involved in Parkinson’s disease (PD). Many of the currently available drugs for the treatment of PD are not sufficiently effective in preventing progress of the disease and have multiple side-effects. With this background, efficient drug candidates, sulfated polysaccharides from *Chlamydomonas reinhardtii* (Cr-SPs) were isolated and investigated for their effect on inhibition of α-Syn fibrillation and dissolution of preformed α-Syn fibrillar structures through a combination of spectroscopic and microscopic techniques. The kinetics of α-Syn fibrillation demonstrates that Cr-SPs are very effective in inhibiting α-Syn fibrillation. Sodium dodecyl sulphate-polyacrylamide gel electrophoresis gel-image shows presence of soluble protein in the presence of Cr-SPs after completion of the fibrillation process. The morphological changes associated with fibrillation monitored by transmission electron microscopy showed that Cr-SPs efficiently bind with α-Syn and delay the conversion of α-helical intermediate into β-sheet rich structures. Cr-SPs are also effective even if onset of α-Syn fibrillation has already started and they also have the ability to dissolve pre-formed fibrils. Thus, the current work has substantial therapeutic implications towards unlocking the immense potential of algal products to function as alternative therapeutic agents against PD and other protein aggregation related disorders.

## Introduction

Protein misfolding and aggregation/fibrillation are associated with a number of degenerative diseases^1^. Soluble monomers of amyloidogenic proteins can self-assemble and form fibrils under aggregation-prone conditions. Beginning of protein fibrillation *in vivo* is not well understood; however, it is believed that combination of various physiological factors are responsible for protein aggregation/fibrillation. It is also reported that these protein aggregates are either resistant to degradation or escape from the protein degradation pathways due to some aberrations; hence they persist in cells once the initial aggregates are formed^2,3^.

Parkinson’s disease (PD) is the second-most common neurodegenerative disorder after Alzheimer’s disease (AD). It is an aging-related movement disorder and characterized by typical motor symptoms including tremor, inflexibility, and hypokinesia; and non-motor symptoms such as dementia, sleep disorders, depression, responsive, mental, and behavioural disorders. A thorough literature review suggests that PD is strongly coupled to the conformation and aggregation of a small protein α-synuclein (α-Syn)^4–6^. α-Syn is a 140-amino acid containing highly conserved and neuron-specific presynaptic protein, which is encoded by a single gene located in chromosome 4^7^. It is a natively unfolded protein and remains in random coil conformation due to presence of low hydrophobicity and excess negative charge of α-Syn at neutral pH (pI = 4.7)^8^. Nonetheless it can adopt a number of different conformations depending on conditions and cofactors^9^. Due to existence of α-Syn in random coil conformations, covalent modifications such as Ser129 phosphorylation and hydrophobic interactions^10^ initiate the polymerization of various α-Syn proteins into a β-sheet conformation^11^ which gets further intensified by forming linear and lateral hydrogen bonds and results in fibril formation. The accumulation of α-Syn fibrils is sited at the presynaptic terminals, which leads to pathological impact on synaptic function. Moreover, this may result in the loss of dendritic spines at the postsynaptic area. The aggregation of α-Syn is a critical step in the pathogenesis of PD, and the association between α-Syn and PD is supported by several evidences^12,13^.

The most common therapy for PD is based on long-term dopamine (DA) replacement with 3, 4-dihydroxy-L-phenylalanine (L-DOPA), the precursor of DA; although, this therapy is associated with various side effects^14^. Most of the currently available drugs for the treatment of PD are either synthetic or obtained from terrestrial-based natural products^15–17^. The sustained failure of conventional drugs in preventing the progress of the disease, coupled with multiple adverse side-effects have driven researchers to look for efficient drug candidates from alternate natural resources. Marine ecosystem produces a very rich source of potential natural compounds with a broad range of unique pharmaceutical activity. These marine reservoirs such as marine plants, animals and microbes produce various bioactive compounds which have analgesic, anti-cancer, anti-infective, anti-inflammatory, immuno-modulatory, anti-viral, neuroprotective properties^18–22^. One of the recent studies has demonstrated that the extract of marine alga *Alaria esculenta* has ability to modulate α-syn folding and amyloid formation^23^.

It is known that many marine algae species contain SPs and their lower molecular weight oligosaccharide derivatives which are biocompatible, biodegradable and have been shown to offer numerous health benefits. These algal SPs have high nutritional value and pose anti-malaria, anticoagulant, anti-inflammatory, anti-viral, antiparasitic, antioxidant, anti-thrombotic and antilipidemic properties^24–32^. A thorough search in literature suggests that even though efforts have been made to understand the therapeutic potentials of SPs, their therapeutic potential against protein fibrillation associated diseases has not been explored well. In the present work, we have induced and isolated Cr-SPs from green chlorophyte *Chlamydomonas reinhardtii* and evaluated its potential for modulation of α-Syn aggregation using a combination of different spectroscopic and microscopic tools. The current work is done with the objective of unlocking the immense potential of Cr-SPs to act as alternative therapeutic agents for prevention of PD and provide a scientific basis for the development of new generation of phytopharmaceuticals.

## Results and Discussion

### Analysis of chemical composition of algal sulfated polysaccharides

The total carbohydrate content extracted from ethanol was found to be ~68%, reducing sugars 15.93% and non-reducing sugars 52.07%. The protein and the sulphate contents were estimated to be 1.8% and 29.4%, respectively. These results clearly indicate that the extract is enriched with sulfated polysaccharides. Also the FTIR analysis of the Cr-SPs showed characteristic side chains corresponding to sulfated polysaccharides^33^. Similarly earlier reports in *Spirulina plantesis*, showed that extraction of sulfated polysaccharides with ethanol gave significantly more carbohydrate content than water extraction^34^. However the amount of carbohydrate content produced by *Chlamydomonas reinhardtii* is much more than *Spirulina plantesis* carbohydrate content which is 13.16% with ethanol extraction method.

### α-Synuclein (α-Syn) fibril formation

**Figure 1:**
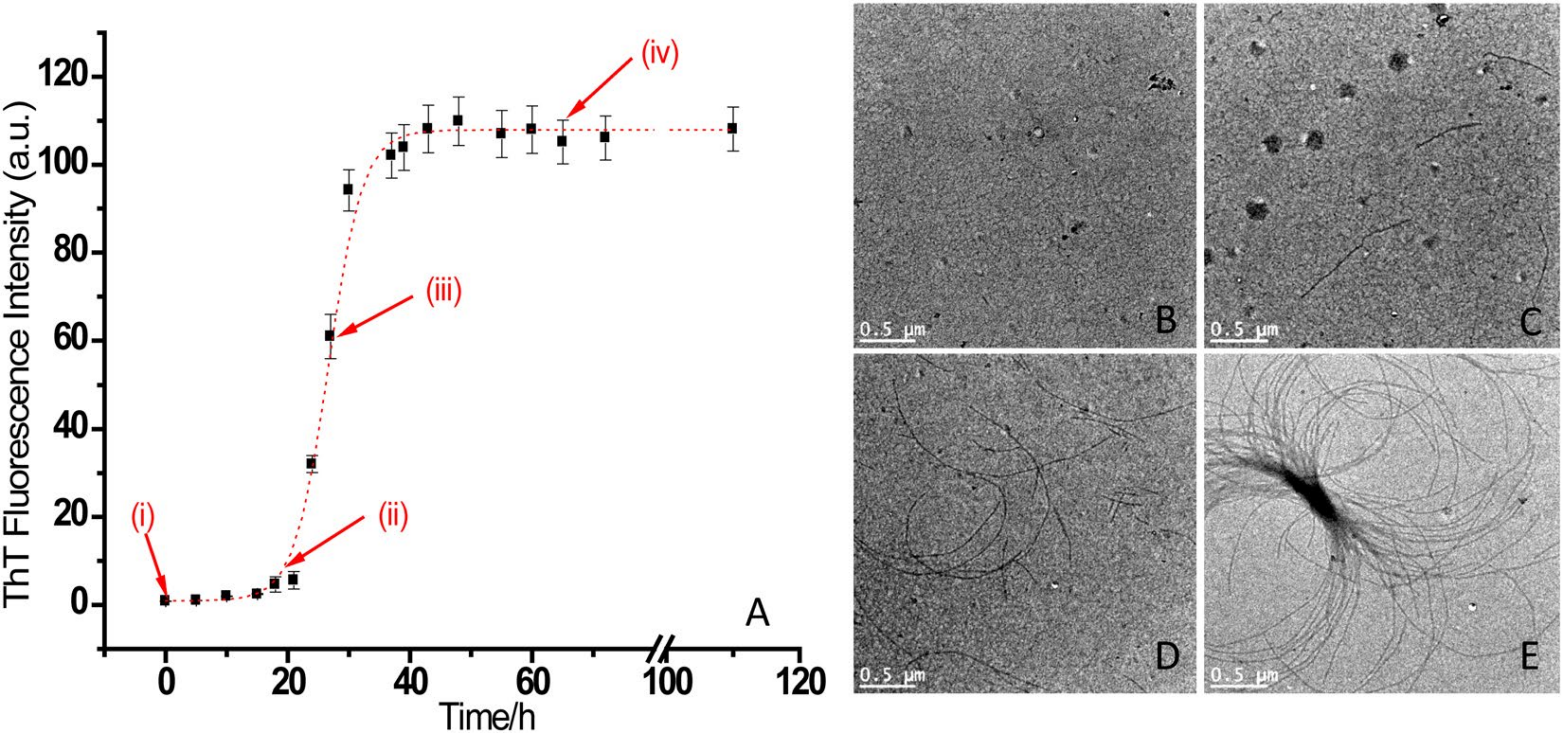
(**A**) Kinetics of the α-Syn fibrillation monitored by ThT binding assay, and transmission electron microscopic (TEM) images of α-Syn fibrils at (**B**) 0 h, (**C**) 21 h, (**D**) 28 h and (**E**) 65 h of the fibrillation process.

Figure 1A shows the time course of fibrillation of α-Syn when incubated at pH 7.4 and 37 °C stirred at 68 rpm. The fibrillation process was monitored by ThT binding assay. The fibrillation curve shows sigmoidal behaviour consisting of three distinct phases; initial lag phase, a subsequent elongation phase and a final saturation phase. The plots exhibit lag phase kinetics and fit well to a sigmoidal function described by equation (1) (R^2^ = 0.995 to 0.985). This confirms that α-Syn fibrillogenesis follows nucleation-dependent polymerization model of aggregation^35^. The lag time (t) and apparent growth rate constant (k_*app*_) for α-Syn fibrillation were calculated by using equation (1) and are found to be (21 ± 1) h and (0.4 ± 0.1) h^−1^ respectively. The α-Syn fibrillation process was also studied by performing transmission electron microscopy (TEM) in a time dependent manner. The images were acquired at 0 h, 21 h, 28 h and 65 h of the fibrillation process which correspond to points (i), (ii), (iii) and (iv) of the Fig. 1A and are shown in Fig. 1B–E, respectively. The Fig. 1B shows TEM image of native α-Syn which was taken at the beginning of the fibrillation process. After 21 h, the oligomeric structures of α-Syn are formed (see Fig. 1C) which provide nucleus for further extension of fibrils, as shown in Fig. 1D corresponding to incubation for 28 h. After 65 h, long fibrils of α-Syn were observed as shown in Fig. 1E.

### Effects of SPs on α-Syn fibrillation

**Figure 2:**
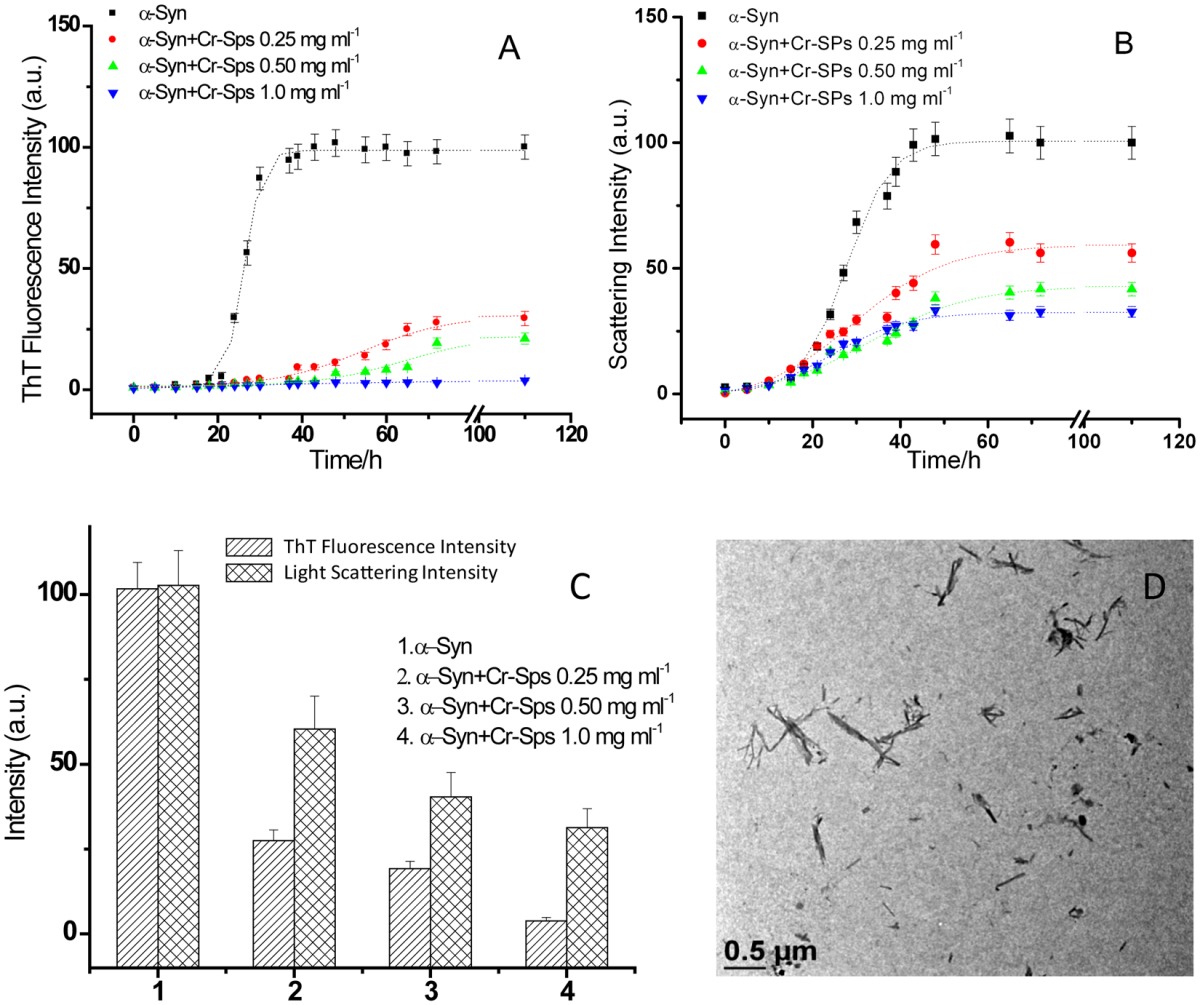
Kinetics of α-Syn fibril extension in the absence and in presence of different concentration of Cr-SPs studied by monitoring the changes in (**A**) ThT fluorescence emission intensity, (**B**) 90° light scattering intensity and (**C**) amplitude obtained from ThT fluorescence and scattering intensities and (**D**) TEM image of α-Syn in presence of 1 mg ml^−1^ Cr-SPs after 65 h of the fibrillation process.

In order to check the effects of Cr-SPs on α-Syn fibrillation, ThT binding assays were performed. The α-Syn solutions were incubated at 37 °C under fibrillation conditions. Figure 2A represents the ThT fluorescence kinetics plots of α-Syn in the absence and presence of different concentrations of Cr-SPs. In the absence of Cr-SPs, ThT intensity did not increase up to 20 h after which it increases sharply upto 42 h beyond which it leads to saturation. The lag time and apparent growth rate constant for α-Syn fibrillation are (21 ± 2) h and (0.4 ± 0.1) h^−1^ respectively. In presence of 0.25 mg ml^−1^ Cr-SPs the ThT fluorescence intensity does not show significant increase even after 40 h of incubation. The lag time (τ) is observed to increase to (33 ± 3) h with a decrease in K_*app*_ (0.1 ± 0.04) h^−1^ in the presence of 0.25 mg ml^−1^ Cr-SPs. With an increase in the concentration of Cr-SPs to 0.50 mg ml^−1^, the lag time further increased to (44 ± 3) h. The kinetic parameters for α-Syn fibrillation could not be calculated in the presence of 1 mg ml^−1^ Cr-SPs as the ThT fluorescence is quenched almost completely.

The ThT assays alone are not enough to indicate the extent of fibril formation in the fibrillation process^36^. Therefore, to ascertain the inhibition of α-Syn fibrillation by Cr-SPs, 90° light scattering measurements were done, which provide the molecular sizes the aggregates formed. Figure 2B shows plots of light scattering for α-Syn in the absence and presence of different concentrations of Cr-SPs. Similar to ThT assay, when the concentration of Cr-SPs is increased, the scattering intensity decreases as a function of their concentration. It is also observed that the lag time calculated by using equation (1) is not affected significantly when the concentrations of Cr-SPs is increased. The ThT fluorescence is known to detect the fibrillar aggregates whereas light scattering identifies both the fibrillar as well as the amorphous aggregates. The results suggest that Cr-SPs have delayed the onset of α-Syn fibrillation whereas the onset of formation of amorphous aggregates during the aggregation process remains unaffected.

The amplitude obtained from the data fitting of ThT fluorescence and scattering intensities to equation (1) signifies the extent of α-Syn fibrillation. The suppression of the extent of α-Syn fibrillation by Cr-SPs is clearly reflected in terms of decrease in the amplitude of ThT fluorescence and the scattering intensities with increase in Cr-SPs concentration (see Fig. 2C). To ascertain this, transmission electron microscopy of α-Syn in presence of 1 mg ml^−1^ Cr-SPs was performed. Figure 2D shows transmission electron microscopic (TEM) image of α-Syn taken after 65 h of incubation in the presence of 1 mg ml^−1^ Cr-SPs. The TEM image of α-Syn showed a mixture of tiny fibrillar and amorphous aggregates. The α-Syn fibrils formed in this condition are much smaller than those formed in the absence of Cr-SPs and moreover the amount of the fibrils formed also reduces significantly. The reduction in the ThT fluorescence intensity observed in Fig. 2A (blue line) is associated with decrease in length as well as amount of α-Syn fibrils, whereas, the presence of amorphous aggregates is responsible for the occurrence of slight scattering intensity (Fig. 2B, blue line). The above results clearly demonstrate that Cr-SPs exhibit inhibitory effect on α-Syn fibrillation. It has been reported earlier that mammalian sulfated polysaccharides (glycosaminoglycans, chondriontin sulphate, heparin) show neuroprotective potential by reducing the sustenance of oligomeric species^37–42^. However, the Cr-SPs used in the current study are from plant source and the exact mechanism by which these offer neuroprotection needs to be understood.

### Exposed hydrophobic surfaces of α-Syn in presence of Cr-SPs

When a protein undergoes fibrillation, initially it becomes partially unfolded to a slightly looser conformation as compared to the native state. Therefore it is expected to have greater exposed hydrophobic groups in the partially unfolded conformation^43^. This facilitates association of more monomers of similar kind of structures which finally results in the formation of aggregates^44^. 1-Anilino-naphthalene-8-sulfonate (ANS) is a fluorescent probe which is widely used to study protein folding/unfolding^45^. ANS gives emission maximum at 500 nm when present inside aqueous environment but shows blue shift and intense fluorescence emission when bound to hydrophobic patches of the protein^46^. The extent of blue shift and emission intensity vary with the structure of protein environment around ANS.

**Figure 3:**
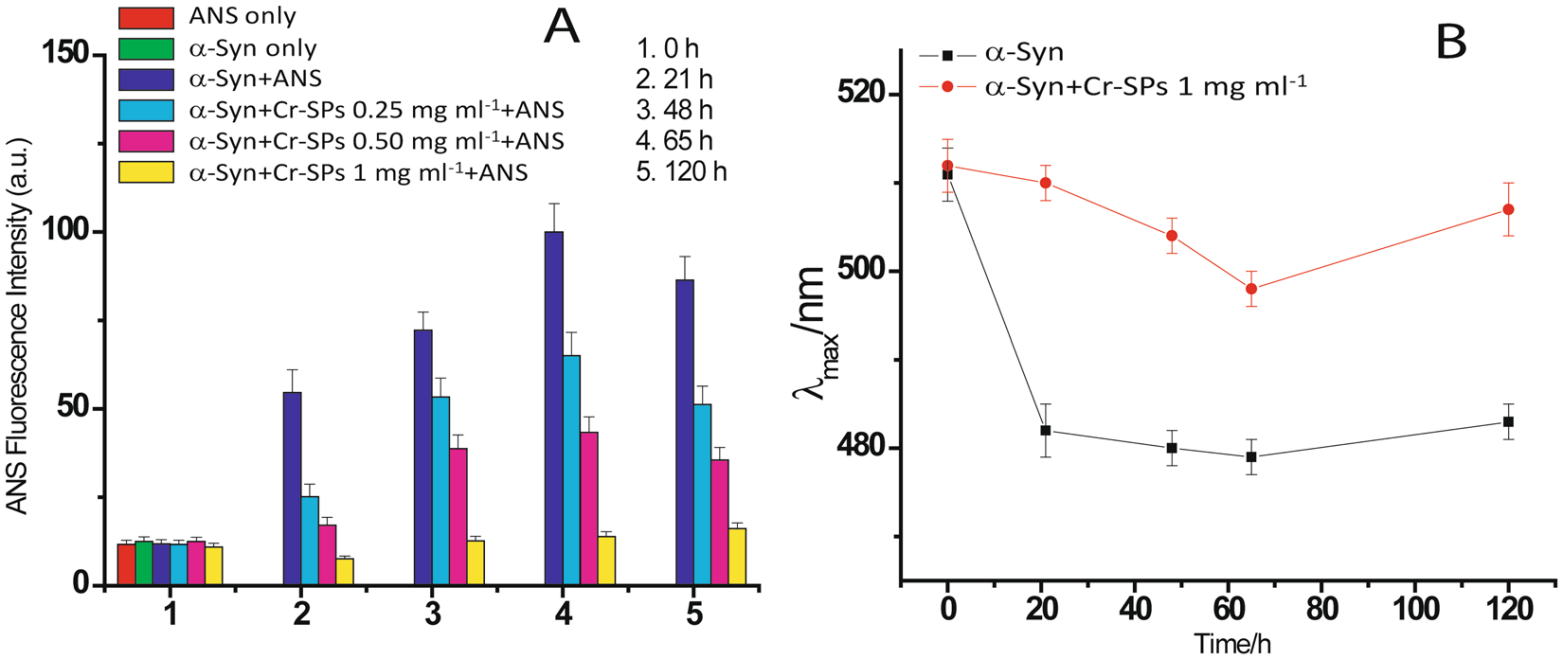
(**A**) Maximum ANS fluorescence intensity in the absence and presence of different concentrations of Cr-SPs and (**B**) blue shift of λ_max_ of ANS fluorescence in the absence and presence of 1 mg ml^−1^ Cr-SPs at different time intervals of α-Syn fibrillation.

Figure 3A shows maximum ANS fluorescence intensity in the absence and presence of different concentrations of Cr-SPs at different time intervals of α-Syn fibrillation. Initially at 0 h, ANS shows very weak fluorescence emission in the absence and presence of different concentrations of Cr-SPs. This is because α-Syn in its native conformation is a random coil and hence there are no sites for binding of ANS. After an incubation for 21 h, α-Syn molecules are in the oligomeric state after crossing the nucleation phase of the fibrillation process, thereby leading to the formation of some binding sites for ANS. Consequently, an increase in the ANS fluorescence intensity is observed with a blue shift of (30 ± 3) nm (Fig. 3B). As the α-Syn fibrillation proceeds, more and more binding sites are formed which facilitates binding of more ANS molecules. The fluorescence intensity of ANS continues to increase with an incessant blue shift up to 65 h after which a slight drop in the ANS fluorescence intensity was observed (at 120 h). This could be attributed to relatively lesser availability of hydrophobic patches when α-Syn has formed mature fibrillar aggregates.

Interestingly it was observed that the rise in the ANS fluorescence decreases in the presence of Cr-SPs in a concentration dependent manner. In the presence of 1 mg ml^−1^ Cr-SPs, ANS fluorescence did not increase significantly during α-Syn fibrillation. It is evident from Fig. 3A that Cr-SPs either do not allow the formation of binding sites for ANS which are mainly hydrophobic patches or bind at the same site where ANS binds. Both situations will lead to decrease in fibrillation since Cr-SPs do not allow formation of hydrophobic patches which is a key step in the aggregation process. On the other hand if Cr-SPs bind at the same cleft where ANS molecules bind i.e. hydrophobic patches, it is an indication that Cr-SPs interfere in the hydrophobic interaction which is the main driving force for protein-protein association.

### Determination of soluble protein

**Figure 4:**
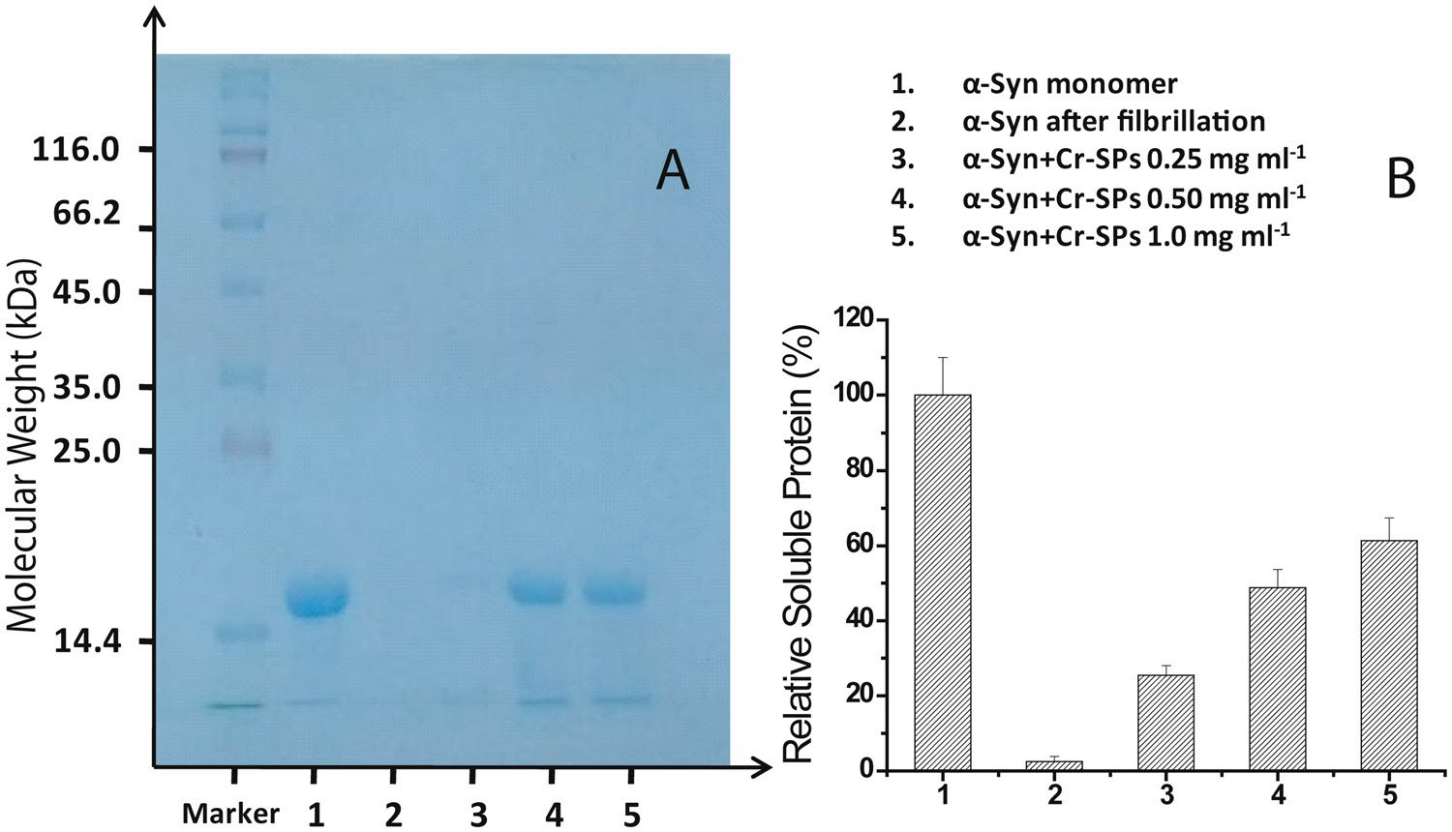
(**A**) SDS PAGE gel image showing soluble protein and (**B**) percent relative soluble protein at the saturation phase of α-Syn fibrillation process (at 65 h).

During a typical protein fibrillation event, soluble monomeric protein gets converted into insoluble fibrillar aggregates. In order to measure the amount of soluble protein remaining at the end of fibrillation, sodium dodecyl sulphate-polyacrylamide gel electrophoresis (SDS-PAGE) of the protein samples incubated in the absence and presence of Cr-SPs was performed. Figure 4A shows the SDS-PAGE gel image of the supernatant of the protein samples which were collected after 65 h of incubation and centrifuged. It is clearly seen from the gel image that the intensity of the protein bands rises with increase in the amount of Cr-SPs. The data presented in Fig. 4B shows a relative increase in the amount of fibrils with increase in the concentration of Cr-SPs. Figure 4 clearly demonstrates that Cr-SPs prevent conversion of soluble α-Syn into insoluble aggregates and hence inhibit α-Syn fibrillation.

### Kinetics of seeded fibrillation

The onset of PD *in vivo* is not very clear; however, it is suggested that the presence of misfolded proteins/small aggregates acts as seeds to induce misfolding and aggregation of nascent polypeptides^47^. Seeding is responsible for acceleration of *in vivo* aggregation of soluble monomers, hence has important implications in the spreading of PD^48–50^. Therefore, the ability of α-Syn aggregates which are formed in presence of Cr-SPs to act as seeds were then checked. In case of seeded aggregations, the nucleation phase is bypassed leading to spontaneous exponential fibrillation of the soluble monomers^51^.

**Figure 5:**
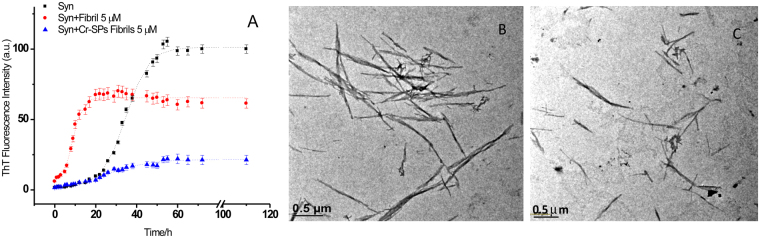
(**A**) ThT fluorescence representing the kinetics of α-Syn fibrillation in the absence [■] and in the presence of seeds formed without Cr-SPs [●], and seeds formed with Cr-SPs [▲]. Figures (**C**) and (**D**), respectively show TEM images of α-Syn fibrils taken after 65 h of incubation in the presence of seeds formed without 1 mg ml^−1^ Cr-SPs and with 1 mg ml^−1^ Cr-SPs, respectively.

Figure 5A represents ThT binding profiles for seeded aggregations of α-Syn. *In vitro* seeded experiments can be considered as oversimplified models of *in vivo* aggregation^52^. For all the seeded experiments of α-Syn fibrillation, the concentrations of α-Syn monomers and preformed fibrils (seeds) were 250 μM and 5 μM, respectively. Here also the kinetics parameters such as lag time and growth rate constant were calculated using equation (1) which are (4 ± 1) h and (0.5 ± 0.1) h^−1^, respectively. Seeding has accelerated the fibrillation process with increase in the growth rate constant and five-fold decrease in the lag time compared to the non-seeded experiments. In order to visualise the fibrils formed in the seeding experiments, the TEM imaging was then performed. Figure 5B shows the TEM image of α-Syn fibrils formed during the seeding experiments. Here the morphology of the α-Syn fibrils is quite different from that formed without seeds (see Fig. 1D). The fibrils are shorter in length and show branching. The solid state NMR studies suggest that physicochemical and conformational compatibility between seeds and monomers can influence the morphological features of the α-Syn fibrils^53^.

When seeding aggregation was performed using preformed fibrils (formed in presence of 1 mg ml^−1^ Cr-SPs) as seed, the values of lag time and growth rate constant are (17 ± 3) h and (0.04 ± 0.02) h^−1^ respectively. However, the amplitude of fibrillation decreased significantly to (10 ± 3) a.u. The elongation of the seeds in the case of α-Syn aggregation is proposed to occur via addition of monomers to the elongating ends^54^. The extent of fibrillation and the rate of elongation depend on affinity and conformational compatibility between seeds and monomers^55^. The results suggest that the fibrillar species which are formed in the presence of Cr-SPs do not act as seeds for secondary nucleation reactions. The 90° light scattering intensities of aggregates were then measured and it was observed that the fibrillar aggregates formed in the presence of seeds have lesser intensity (128 ± 7) compared to those formed in the absence of seeds (255 ± 10). However, the aggregates formed in the presence of seeds which are formed in the presence of Cr-SPs showed least intensity (73 ± 5). Therefore the observed reduction in the ThT intensity can be rationalized in terms of non-compatibility between the seeds and the monomers which results in lesser fibrillation of α-Syn or formation of some amorphous aggregates that are unable to bind to ThT. For further verification, the TEM imaging was performed. The TEM image of the sample showed a mixture of α-Syn fibrils and amorphous aggregates (Fig. 3C). The fibrils formed under this condition are branched and small in length.

### Effects of intermittent addition of SPs on α-Syn fibrillation

**Figure 6:**
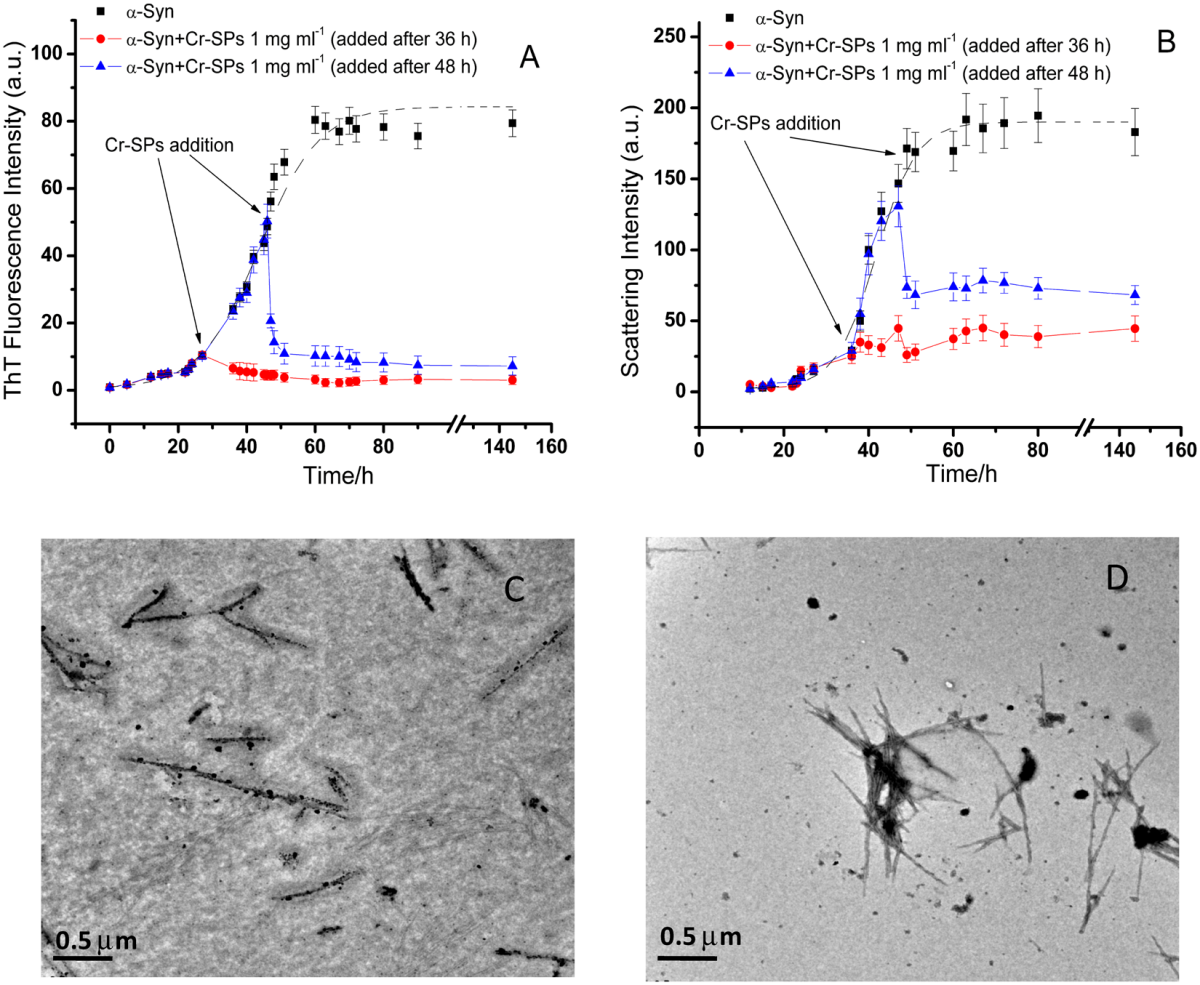
Kinetics of α-Syn fibrillation upon intermittent addition of 1 mg ml^−1^ Cr-SPs after 36 h [●] and 48 h [▲] monitored by ThT fluorescence emission intensity (**A**) and 90° light scattering intensity (**B**). Figures (**C**) and (**D**), respectively show the TEM images of α-Syn taken upon addition of 1 mg ml^−1^ Cr-SPs after 36 h and 48 h of incubation. These images were taken after 65 h of total incubation period.

The kinetics of α-Syn fibrillation after intermittent addition of Cr-SPs was also studied in order to see the effects of Cr-SPs on α-Syn before and after the start of fibrillation. The Cr-SPs were added to the α-Syn samples undergoing fibrillation process at two stages (i) when the elongation phase had just started (at 36 h) and (ii) in the mid-way of the elongation stage (at 48 h) of fibrillation. Figure 6A shows the ThT binding profile of α-Syn in the absence (black line) and in the presence of Cr-SPs at 36 h (red line) and 48 h (green line) addition. The Cr-SPs addition just after the nucleation phase (at 36 h) does not lead to increase in the ThT emission intensity further. This was also confirmed by 90° light scattering kinetics experiments (Fig. 6B, red line). A possible reason is that the presence of Cr-SPs has arrested the elongating fibrils in that stage only without further attachement of the monomeric protein to the elongating fibrils. To understand it further, the TEM image of the samples were recorded. Figure 6C shows the TEM image of α-Syn fibrils recorded after 65 h of incubation. The image clearly shows that the Cr-SPs have bound to the growing fibrils and formed a coating sheath around them. The presence of sheath like structures further prevent attachment of monomers to the growing ends thereby inhibiting further elongation.

Interestingly, when Cr-SPs were added after 48 h of incubation (at middle of the elongation phase), the ThT intensity dropped significantly and a similar kind of pattern was observed with 90° light scattering intensity as well. To know more about this, transmission electron microscopy of α-Syn was performed. The TEM image taken after 65 h of incubation shows that the addition of Cr-SPs to the α-Syn fibrils in their elongation phase results in association of protein molecules to form bundle like structures, covered with the sheath of Cr-SPs. This leads to seizure of elongating fibrils on one hand and decrease in ThT intensity (ThT is unable to bind with fibrils) on the other hand. The above results suggest that Cr-SPs interfere efficiently in the fibrillation process even when the onset of aggregation/fibril elongation of α-Syn has already started.

### Effects of Cr-SPs on secondary structure of α-Syn during fibrillation

**Figure 7:**
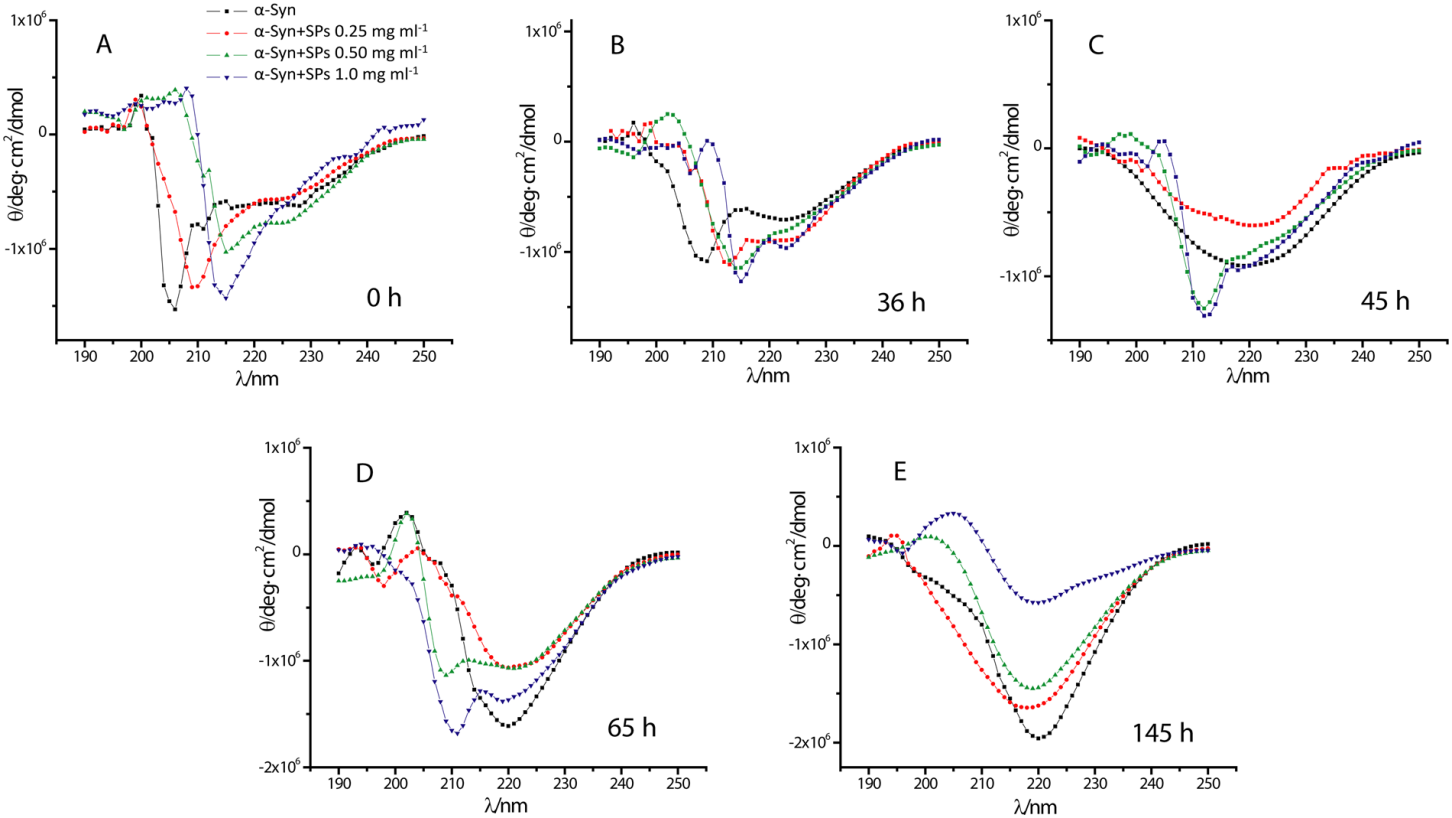
Far UV-CD spectra of α-Syn in the absence and presence of different concentrations of Cr-SPs at (**A**) 0 h, (**B**) 36 h, (**C**) 45 h, (**D**) 65 h and (**E**) 145 h of the fibrillation process.

Protein fibrillation is driven by large conformational alterations where different secondary structural components are converted to β-sheet rich structure. To know about the effect of Cr-SPs on the secondary structure of α-Syn during fibrillation process, circular dichroism (CD) spectroscopy was performed at different time points of fibrillation process. Figure 7A shows the far-UV CD spectra of α-Syn in the absence and presence of different concentrations of Cr-SPs at the beginning of the fibrillation process (0 h). At this time point, α-Syn is in its native state and shows random coil conformation (black line). The far UV CD spectra of α-Syn in the presence of different amounts of Cr-SPs show shift in negative peaks (Fig. 7A: red, green and blue lines). This suggests binding of Cr- SPs to α-Syn induces conformational changes in the protein. It has been reported that algal extract has ability to bind and modulate α-Syn folding and aggregation^56^. Steady state fluorescence measurements on binding of Cr-SPs with α-Syn were also performed (Fig. S1). Addition of Cr-SPs causes significant quenching of tyrosine fluorescence due to binding and conformational changes in α-Syn.

Figure 7B shows CD spectra of α-Syn at 36 h of the fibrillation process. At this time point α-Syn is in the nucleation stage and is expected to be in the oligomeric state. During fibrillation, the oligomeric species of α-Syn are reported to form α-helical structures^57^, which remain there for a short time. The far UV-CD spectra of α-Syn in the absence of Cr-SPs (black line) showed well-defined negative peaks at 210 and 222 nm which corroborates well with the earlier reports^57^. However, similar spectra also arise from a combination of random coil and β-sheets during the random coil to β-sheets transition. As the fibrillation precedes (after 45 h), the negative peaks at 210 and 222 nm disappear and a single minimum arises at ~218 nm (Fig. 7C, black line) which is characteristic of β-sheets^58,59^. At this time point α-Syn is in its elongation phase and is expected to have β-sheets rich structures. It is also observed that in the presence of 0.25 mg ml^−1^ Cr-SPs, β-sheets have started appearing but to a lesser extent as compared to that in their absence (Fig. 7C, red line). On the other hand in the presence of 0.50 mg ml^−1^ and 1 mg ml^−1^ Cr-SPs, still significant α-helical content can be seen (Fig. 7C, blue and green lines). This suggests that the presence of Cr-SPs stabilize α-Syn and prevent conversion of α-helical structures into β-sheets. Similar kind of spectra were obtained even after 65 h of fibrillation process (see Fig. 7D) up to which Cr-SPs are able to prevent conversion of α-helical structures into β-sheets and hence delay the fibrillation process.

After 145 h of incubation when the fibrillation process is at saturation stage, the formation of β-sheets are seen in all the cases but the amount of β-sheets formed varies (Fig. 7E). In the absence of Cr-SPs, the CD spectra of α-Syn shows negative ellipticity at ~220 nm which continues to decrease in the presence of Cr-SPs upto a concentration corresponding to 1 mg ml^−1^ (Fig. 7E). This suggests that even though the fibrils are formed in all the cases, the presence of Cr-SPs (1 mg ml^−1^) suppresses the extent of β-sheets formed.

### Dissolution of α-Syn fibrils by Cr-SPs

**Figure 8:**
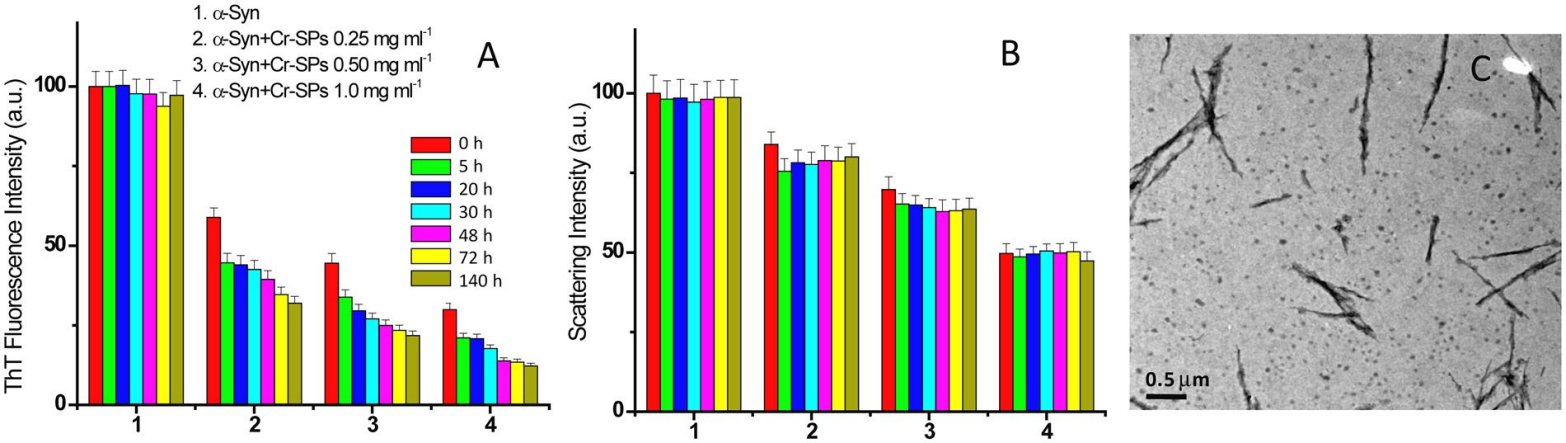
Dissolution of α-Syn fibrils in the absence and presence of different concentrations of Cr-SPs monitored by (**A**) ThT binding assay, (**B**) light scattering measurement as a function of time and (**C**) TEM images of α-Syn fibrils after 72 h of incubation in presence of 1 mg ml^−1^ Cr-SPs.

In order to check the potential of Cr-SPs in dissolving the α-Syn fibrils, kinetics studies were carried out by performing ThT binding assay and light scattering experiments. The fibrils (250 µM) were incubated in the presence of different concentrations of Cr-SPs. Aliquots were taken out from the samples at different time intervals and diluted to a final concentration of 3 µM in order to record ThT fluorescence and light scattering intensities. It is evident from Fig. 8A and B that the presence of Cr-SPs decreases the ThT fluorescence intensity significantly whereas the decrease in the scattering intensity is much lesser. The ThT fluorescence emission intensity is more sensitive to the presence of fibrils than amorphous aggregates. On the contrary, the scattering data indicates the molecular dimensions of the species present.

The TEM imaging was also done for further verification of the above observations (see Fig. 8C). The TEM images for dissolution of α-Syn fibrils were recorded after 72 h of incubation in presence of 1 mg ml^−1^ Cr-SPs. It is seen that in the presence of 1 mg ml^−1^ Cr-SPs, long fibrils of α-Syn have been broken/dissolved into smaller fibrils (Fig. 8C). This clearly demonstrates that Cr-SPs are not only capable of inhibiting α-Syn fibrillation but also dissolve preformed fibrils effectively.

Glycans from marine origin have variable chain length and contain sulfate at peculiar positions, which provides different degrees of flexibility, orientation, and hydrophobicity to them. These features are extremely important for a molecule to act like a drug and provide improved therapeutic properties. They are capable of binding to a range of proteins with high specificity and are involved in cell signaling, cell development, cell adhesion, cell differentiation and cell matrix interactions. Sulfated polysaccharide from the brown algae *Laminaria japonica* have been reported to exhibit neuroprotective effect by decreasing apoptosis in PC12 neuronal cells^60^. It is also reported that the isolated sulfated polysaccharides may competitively inhibit binding of Aß with intrinsic heparan sulfate glycosaminoglycan, and prevent amyloid formation^61^. They also have the ability to bind and modulate α-Syn fibrillation. The sulfated polysaccharides may be further screened for their abilities to function as glyco-mimetics that may prevent the aggregation and neurofibrillar formation, thereby opening novel avenues towards therapy and management of PD. The results obtained in this work suggest that the use of algal Cr-SPs could serve as an efficient alternative approach for the therapy and management of synucleopathies and other protein aggregation related diseases.

## Conclusions

A combination of spectroscopy and microscopy has demonstrated the potential of algal sulfated polysaccharides (SPs) in the prevention of α-Syn fibrillation. The use of ThT as a fluorescent probe along with the 90° light scattering experiments has successfully enabled elucidation of the lag period and extent of fibril formation in the protein solution. The Cr-SPs are observed to interfere in α-Syn fibrillation even if the fibrillation has already started. The data also suggest that the fibrillar aggregates formed in the presence of Cr-SPs do not act as seeds and hence do not serve as templates for secondary nucleation. The results have effectively established that Cr-SPs are capable of dissolving the preformed α-Syn fibrils. To the best of our knowledge this is the first concrete report showing the ability of Cr-SPs to act as an alternative therapeutic agent for prevention of protein fibrillation/aggregation related disorders.

## Materials and Methods

### Cell culture and growth conditions

*C. reinhardtii* (strain CC-124) was obtained from the *Chlamydomonas* Genetic Center, Duke University, USA. Cells were grown and maintained by periodic transfers in Tris Acetate Phosphate (TAP; pH 7) as mentioned by Sirisha *et al*.^62^.

### Extraction and purification of sulfated polysaccharides from *Chalmydomonas reinhardtii*

For polysaccharide extraction, algae was cultured in 3 L autoclaved TAP medium containing 30 mM NaCl and incubated at 25 ± 3 °C incubator shaker. The cultivated flasks were illuminated for 24 h with continuous cool white fluorescent lamps. The algal cells once attained stationary phase, they were pelleted down by centrifuging at 1100 × *g* for 5 min. The cell pellet was crushed with a mortar and pestle, suspended in 80% ethanol, and transferred to water bath at 80°C for 4 h. After 4 h, the extract was allowed to cool to room temperature and centrifuged at 4000 × *g* for 10 min to remove debris. The supernatant was then evaporated under vacuum at 60 °C. The crude extract was then applied onto Q-Sepharose ^TM^ (GE-Health care) fast flow column which is equilibrated with water. The column was eluted step wise with distilled water followed by NaCl gradient and multiple fractions were collected. The semi-purified fractions showing high carbohydrate content were pooled and used for further analysis^33,63,64^.

### Analysis of chemical composition

The total amount of carbohydrate content was estimated by phenol/sulfuric acid reagent according to Dubois *et al*.^65^. Reducing sugars were estimated by dinitrosalicylic acid (DNSA) method according to Miller *et al*. using glucose as standard^66^. The difference between total carbohydrates and reducing sugars were carried out to determine the non- reducing sugar content. Barium-gelatin method was carried out to determine the sulfate content^67^, using sodium sulphate as a standard. The protein content was measured by Bradford’s method^68^, using bovine serum albumin as a standard.

### Expression and purification of α-synuclein

The clone of α-Syn was obtained from Nanobiophysics laboratory, University of Twente, Netherlands. The protein was expressed and purified by following a protocol described by Ghosh *et al*.^57^.

### ThT fluorescence kinetics and 90° light scattering studies for monitoring α-synuclein fibrillation

α-Syn was taken from freshly prepared stock solution and diluted in 10 mM phosphate buffer saline at pH 7.4 containing 100 mM NaCl, to a final concentration of 250 μM. In order to induce fibrillation, α-Syn solutions were incubated at 37 °C with rotation at 68 rpm in an incubator rotator procured from Trishul Laboratory Equipments, India. All the kinetics experiments were performed in triplicates with volume of 1000 μL each in 2 mL Lo-Bind round-bottom Eppendorf centrifuge tubes. The fibrillation of α-Syn was monitored by using Thioflavin T (ThT) dye which specifically binds to amyloid fibrils and gives enhanced ThT florescence emission intensity at 480 nm when excited at 450 nm^69^. A stock solution of ThT was prepared in phosphate buffer (10 mM, pH 7.4) and its concentration was determined by using an extinction coefficient *E* = 26,620 M^1^cm^−1^ at 412 nm^70^. At different time intervals an aliquot of incubated sample was mixed with ThT such that the final concentrations of protein and ThT for the fluorescence measurements were 4 µM and 20 µM, respectively. All the fluorescence measurements were done on an Agilent spectrofluorimeter with excitation and emission slit widths fixed at 5 nm each. The samples were excited at a wavelength 450 nm and emission was detected at 480 nm^69^.

For 90° light scattering experiments aliquots were taken out at different time intervals and diluted in phosphate buffer (10 mM, pH 7.4) such that the final concentration of the α-Syn was 4 μM. The excitation wavelength and emission wavelength were fixed at 350 nm for 90° light scattering measurements.

The acquired data from ThT fluorescence and light scattering measurements were analysed by using the following equation^71^.

1 $$Y(t)={Y}_{i}+{m}_{i}t+\frac{{Y}_{f}+{m}_{f}t}{1+{e}^{-[(t-{t}_{0})/\tau ]}}$$

where *Y* is the fluorescence intensity, *Y*_*i*_ and *Y*_*f*_ are initial and final fluorescence intensities, *t* is time, and *t*_0_ is the time to reach 50% of maximal fluorescence. The lag time was determined by *t*_0_ − 2*τ* where *τ* is the time constant of fibril growth and obtained by nonlinear regression.

### ANS binding assay for monitoring exposed hydrophobic surfaces

The experiments on binding of 1-anilino-naphthalene-8-sulfonate (ANS) with α-Syn were carried out on an Agilent fluorescence spectrophotometer with a 1 mL quartz cell of 0.2 cm path length. A stock solution of ANS was prepared in phosphate buffer (10 mM, pH 7.4) and its concentration was determined by using an extinction coefficient of ANS as E_350_ = 5000 M^−1^ cm^−1^ ^72^. The excitation wavelength was set at 365 nm to selectively excite the ANS molecules, and the emission spectra were monitored in the wavelength range of 380–600 nm. The emission spectra of the ANS solutions in buffer were subtracted from those of protein with ANS solutions for blank corrections.

### Sodium dodecyl sulphate-polyacrylamide gel electrophoresis (SDS-PAGE) calculating soluble protein

The samples were collected after attaining saturation period (65 h) of fibrillation process and centrifuged at 3000 rpm for 15 minutes. The supernatant was collected and 20 μl of the samples were dissolved in SDS sample buffer (final SDS concentration 10%), then kept at 90 °C for 5 min before loading the SDS-PAGE gel (15%). The protein bands were visualized using Coomassie R-250 staining. The densitometry of the bands were analysed using the program Image J, version 1.51j, available at http://imagej.nih.gov/ij ^73^.

### Seeding Experiments

Fibrillation reactions were set up by using 5 μM preformed fibrils as seeds and 245 μM α-Syn, at pH 7.4 containing100 mM NaCl. The seeds for aggregation were prepared by sonication of the fibrils in thin-walled PCR tubes for 2 min in a bath sonicator (Branson 1510). The samples for seeding experiments were kept for fibrillation in the similar manner as described in the previous section. The aggregation process was monitored by performing ThT fluorescence and light scattering experiments and measurements were done at different time intervals.

### Transmission Electron Microscopy for studying morphology of α-Syn fibrils

The visualization of α-Syn fibrils was done on a JEOL JEM-2100 Electron Microscope which operates at an accelerating voltage of 200 kV. The TEM samples were prepared by depositing 10 μL of fibril sample diluted 20 times in filtered buffer (10 mM phosphate buffer, 10 mM NaCl at pH 7.4) on Formvar-coated 75 mesh copper grids. The samples were negative stained with 2% aqueous uranyl acetate solution. Uranyl acetate is known to produce high electron density and image contrast as well as impart fine grain to the image^74^. After pre-rinsing with large volumes of water, the grids were dried to acquire images.

### Circular dichroism spectroscopy

The far UV CD spectra (190–250 nm) were recorded to study conformational transition during fibrillation process. At different time intervals an aliquot of incubated sample solution was taken out and diluted in the same buffer so that the final concentration of α-Syn was 10 µM. All the spectra were recorded using a 0.2 cm path length, quartz cuvette with a scan rate of 100 nm min^−1^. Molar ellipticity was calculated from observed ellipticity using following equation:

2 $$[\theta ]=100\cdot (\frac{\theta }{C}\cdot l)$$

where *C* is the concentration of the protein in mol dm^−3^ and *l* is the path length of the cuvette in centimetres. All the spectra were baseline corrected with an average of three accumulations. Two independent experiments were performed in duplicates.

## Electronic supplementary material

Supplementary information
