# Supplementary material for: Unravelling the inhibitory activity of Chlamydomonas reinhardtii sulfated polysaccharides against α-Synuclein fibrillation
Source: Sci Rep. 2018 Apr 9;8:5692. doi: 10.1038/s41598-018-24079-7 (PMC5890252; doi:10.1038/s41598-018-24079-7)
Supplement: Supplementary file 1 — Supplementary information [file 41598_2018_24079_MOESM1_ESM.pdf]

Unravelling the inhibitory activity of *Chlamydomonas reinhardtii* sulphated polysaccharides against α-Synuclein fibrillation.

Sinjan Choudhary*, Shreyada N. Save and Sirisha L Vavilala*

UM-DAE Centre for Excellence in Basic Sciences, University of Mumbai, Kalina Campus, Mumbai 400098, India.

Authors Affiliation

Shreyada N. Save

Junior Project Assistant

Department of Chemistry

UM-DAE Centre for Excellence in Basic Sciences, University of Mumbai, Kalina Campus, Mumbai 400098, India.

Email id: shreyada.save@cbs.ac.in

Corresponding Authors

*Dr. Sinjan Choudhary

Assistant Professor

Department of Chemistry

UM-DAE Centre for Excellence in Basic Sciences, University of Mumbai, Kalina Campus, Mumbai 400098, India.

Email id:sinjan.choudhary@cbs.ac.in

*Dr. Sirisha L Vavilala

Assistant Professor

Department of Biology

UM-DAE Centre for Excellence in Basic Sciences, University of Mumbai, Kalina Campus, Mumbai 400098, India.

Email id: sirisha@cbs.ac.in

Figure S1

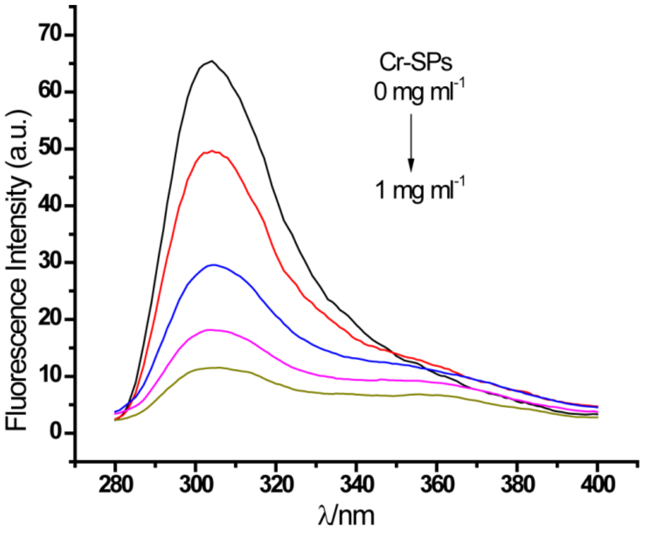

Figure S1: Intrinsic fluorescence spectra of α-Syn in the presence of different concentrations of Cr-SPs.

Figure S2

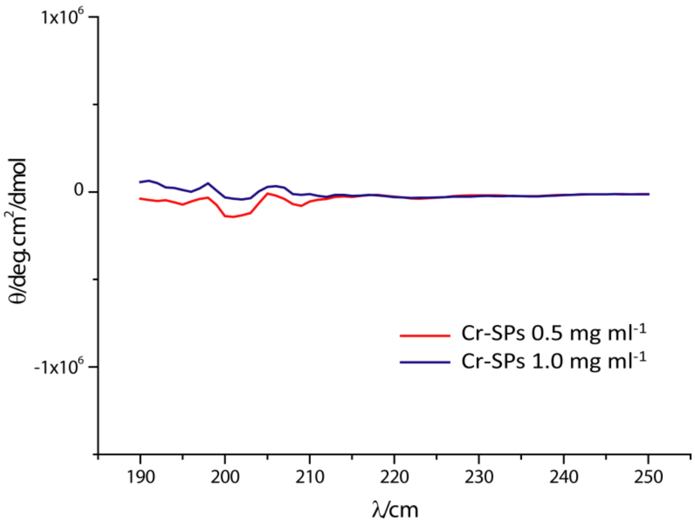

Figure S2: Far UV-CD spectra of Cr-SPs at different concentrations.
